# Theranostic Uses of the Heme Pathway in Neuro-Oncology: Protoporphyrin IX (PpIX) and Its Journey from Photodynamic Therapy (PDT) through Photodynamic Diagnosis (PDD) to Sonodynamic Therapy (SDT)

**DOI:** 10.3390/cancers16040740

**Published:** 2024-02-10

**Authors:** Stuart L. Marcus, Mark P. de Souza

**Affiliations:** SonALAsense, Inc., Berkeley, CA 94710, USA

**Keywords:** aminolevulinic acid, 5-ALA, photodynamic therapy, sonodynamic therapy, photodynamic diagnosis, focused ultrasound

## Abstract

**Simple Summary:**

PpIX, a metabolite of 5-aminolevulinic acid (ALA) is necessary for the synthesis of hemoglobin and cytochromes. ALA has had many clinical uses over the last 33 years. Commercial forms of ALA are used every day along with blue light, which enables the fluorescent detection (PDD) of PpIX, which is specifically accumulated in cancer cells, to find bladder cancers and brain cancers. It has been reported that 12 ALA PD, activated by red light from a laser delivered through fiber optics embedded in brain tumors, can completely clear some brain cancers and increase patient survival. The evolution of ALA PDT to ALA SDT now creates the potential for noninvasive ultrasound to activate PpIX to treat brain cancers as well as cancers almost anywhere in the body through the photodynamic effect. The selectivity of ALA for cancer cells and their accumulation of PpIX should make SDT a low risk procedure.

**Abstract:**

ALA PDT, first approved as a topical therapy to treat precancerous skin lesions in 1999, targets the heme pathway selectively in cancers. When provided with excess ALA, the fluorescent photosensitizer PpIX accumulates primarily in cancer tissue, and ALA PDD is used to identify bladder and brain cancers as a visual aid for surgical resection. ALA PDT has shown promising anecdotal clinical results in recurrent glioblastoma multiforme. ALA SDT represents a noninvasive way to activate ALA PDT and has the potential to achieve clinical success in the treatment of both intracranial and extracranial cancers. This review describes the creation and evolution of ALA PDT, from the treatment of skin cancers to PDD and PDT of malignant brain tumors and, most recently, into a noninvasive form of PDT, ALA SDT. Current clinical trials of ALA SDT for recurrent glioblastoma and high-grade gliomas in adults, and the first pediatric ALA SDT clinical trial for a lethal brainstem cancer, diffuse intrinsic pontine glioma (DIPG), are also described.

## 1. Introduction

Cancer treatment is inherently a multimodal approach, historically centered around surgery, ionizing radiation, and cytotoxic chemotherapy. More recently, targeted therapies, including tyrosine kinase inhibitors [1], immunotherapy [2], various antibodies and antibody-drug conjugates [3], and engineered T-cells [4], have demonstrated potent anti-tumor effects and improved patient outcomes beyond the traditional treatment options. The clear advantage of tumor selectivity of anti-cancer agents is to direct a cytotoxic effect to cancerous cells while sparing healthy tissues, although these therapies typically present their own unique toxicities and off-target effects in patients [1,2,4].

The design of targeted therapies relies on identifying and affecting gene products, pathways, or processes that are selectively altered in tumors. Photodynamic therapy (PDT), photodynamic diagnosis (PDD), and sonodynamic therapy (SDT) seek to take advantage of a quirk in heme metabolism. Exogenously delivered 5-aminolevulinic acid (ALA) results in the accumulation of protoporphyrin IX (PpIX), selectively in tumor cells, which can then be used as an imaging agent (i.e., PDD) or targeted with UV or red and blue light (i.e., PDT) or more recently, focused ultrasound (i.e., SDT), to directly induce cell death. ALA is an endogenous, non-proteinogenic amino acid that is non-toxic and readily distributes throughout the body and crosses the blood–brain barrier. Ultraviolet light has a tissue penetrance of 1–3 mm, which restricts the use of PDD or PDT to the body surface and intraoperative procedures. However, focused ultrasound can deliver energy to deep body tissues, including the brain, which presents a compelling rationale for the development of SDT for brain tumors. Thus, SDT is being developed to specifically target PpIX, which is selectively accumulated in cancerous cells.

### 1.1. The Heme Pathway—Introducing PpIX

**Figure 1:**
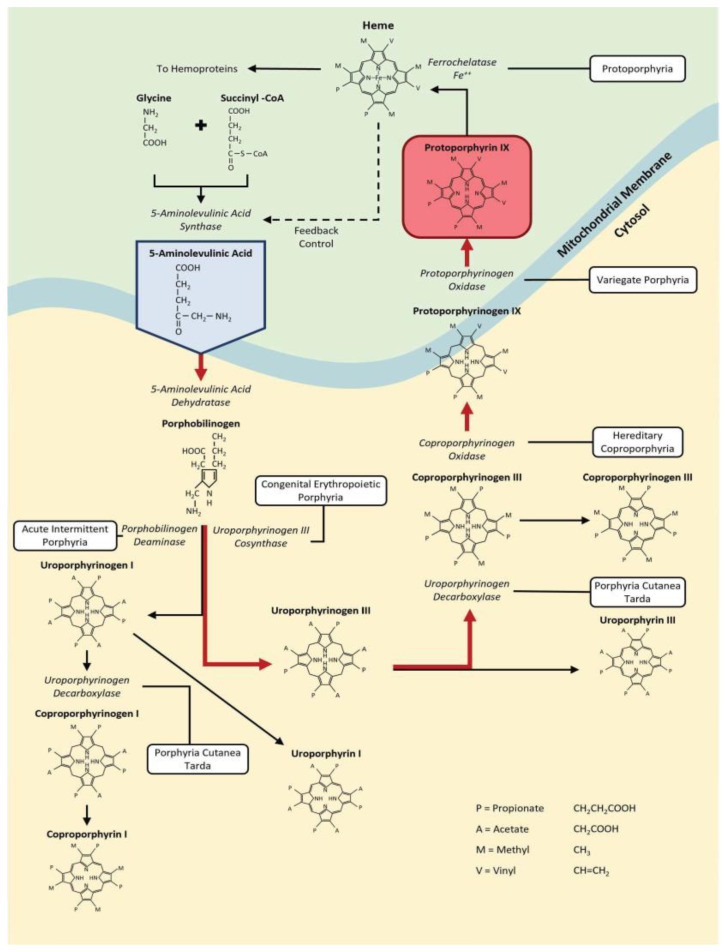
The heme biosynthetic pathway [5]. The red arrows highlight the direct metabolic steps from 5-aminolevulinic acid (in blue box) to protoporphyrin IX (in red box).

Heme is a ferroporphyrin necessary for the functioning of macromolecules like cytochromes, which help the cell utilize energy, and hemoglobin, which is involved in the transfer of molecular oxygen [5]. Heme, although pigmented, is not a photoactive molecule. The final steps in the synthesis of heme take place in the mitochondrion; the pathway is tightly controlled in normal cells by heme, causing feedback inhibition of ALA synthase, the enzyme responsible for the synthesis of 5-ALA (Figure 1).

### 1.2. Erythropoietic Protoporphyria (EPP): Nature’s Experiment in Photodynamic Phototoxicity and Its Use as a Model for ALA PDT

**Figure 2:**
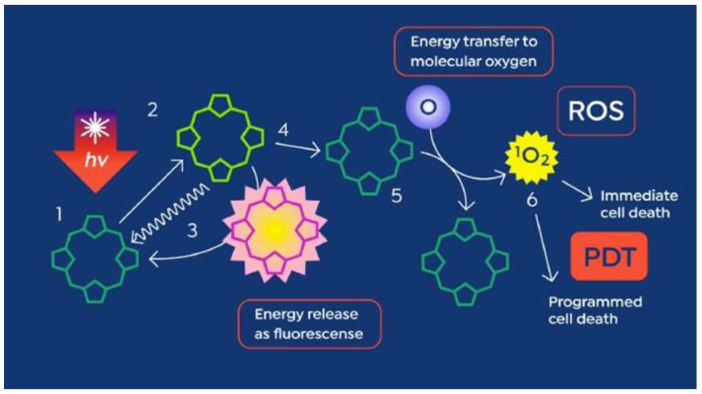
Photoactive properties of PpIX. PpIX is an extremely hydrophobic molecule that binds the plasma membrane and the mitochondrial membrane. Steps 1–3: PpIX absorbs energy from light and releases that energy as fluorescence in light of a longer wavelength than the activating light. Steps 4 through 6 demonstrate the photodynamic effect, in which higher energy PpIX transfers energy to molecular oxygen, creating singlet oxygen (ROS). When used for PDT, adjacent membrane lipid molecules in the plasma membrane or mitochondrial membrane are oxidized, creating cell death via membrane disruption, and programmed cell death (apoptosis) via mitochondrial membrane disruption, cytochrome c release, and activation of the caspase-3 pathway, respectively [6].

EPP is a rare genetic photodermatosis resulting from a partial deficiency of the enzyme ferrochelatase, which is responsible for adding an iron atom to the penultimate molecule in the heme biosynthetic pathway, PpIX [6]. Unlike heme, PpIX is a highly photoactive molecule, capable of strong fluorescence as well as having the ability to participate in photodynamic reactions, in which energy obtained from light is transferred from the PpIX molecule to molecular oxygen (Figure 2), creating singlet oxygen, a short-lived reactive oxygen species.

Due to its hydrophobicity, PpIX accumulates in bile, its natural means of excretion from the body. When PpIX is released in large quantities from the bone marrow in EPP patients into the circulating erythrocytes and plasma LDL, it is taken up by the liver and vascular endothelium, including the superficial skin vasculature and cutaneous nerves, photosensitizing both dermal and epidermal tissue. Accumulated hepatic protoporphyrin can crystallize in hepatocytes and bile canaliculi, causing hepatotoxicity, decreased bile formation and flow, and cholestatic liver failure in some patients [6]. 

Diagnosis of EPP occurs when the infant or young child with EPP is taken out into the sun, which initiates a full-thickness skin phototoxic reaction from PDT and creates excruciating pain, causing the child to cry out until removed from sunlight. Pain may be severe and preceded by a prodrome of tingling, itching, or burning. The pain is not readily relieved by analgesics. Skin damage is seen as erythema and edema of the sun-exposed skin, with blistering also seen in about 26% of cases [6]. The only current treatment for EPP is afamelanotide, a melanocortin 1 receptor agonist that functions as an alpha-melanocyte stimulating hormone analogue to increase production of melanin [7], allowing for attenuation of sunlight so the EPP patient can have greater sun tolerance.

## 2. The Beginning of PpIX Therapeutics—Topical ALA PDT: From Red Light to Blue Light

**Figure 3:**
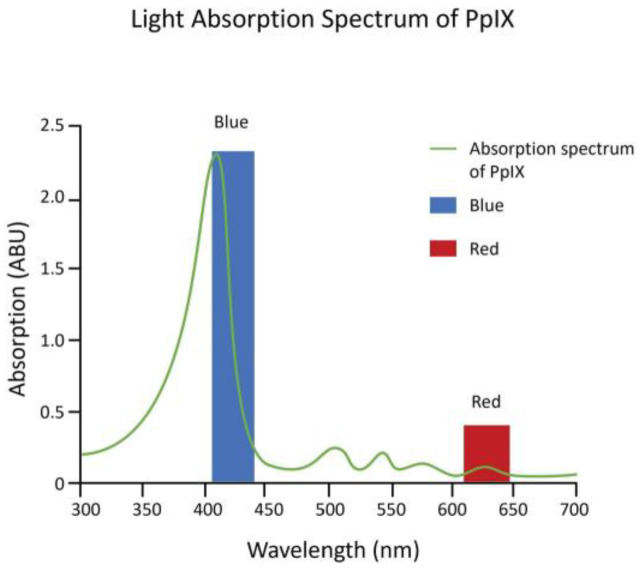
Absorption spectrum of PpIX. The absorption spectrum of PpIX (green line) has the greatest absorption of light within the blue light spectrum (blue bar) vs. the red light spectrum (red bar).

Kennedy et al. (1990) first showed that a state of heightened PpIX accumulation, similar to EPP, could be achieved by applying topical ALA to superficial basal cell carcinomas (sBCCs) under occlusion for 3 h. The accumulation of sufficient PpIX to show robust fluorescence also made possible a therapeutic photodynamic effect using red light from a slide projector, which resulted in a complete tumor response (disappearance of tumor) in 90% of the sBCCs treated as well as actinic keratoses (precancerous lesions) [8]. The results were soon corroborated by investigators using ALA cream formulations and light sources as varied as a slide projector, a copper vapor laser-pumped dye laser, and numerous broadband red light sources [9]. Red light, however, was found to be the least efficient wavelength for activation of PpIX (Figure 3).

While red light was used for the first ALA PDT investigational clinical studies, because red light has the deepest tissue penetration that can still activate PpIX, it only activates the smallest least reactive absorption band (Q band) of the molecule (Figure 3). Therefore, high powers of 50–150 mW/cm^2^ were used, and 40 to 80 J/cm^2^ total light fluence was needed for an effective reaction. Red LED light sources were expensive, and to reduce costs, with the strict power uniformity needed across the surface of the light sources for FDA approval, red lamp working areas were only 4 by 6 inches [10,11].

**Figure 4:**
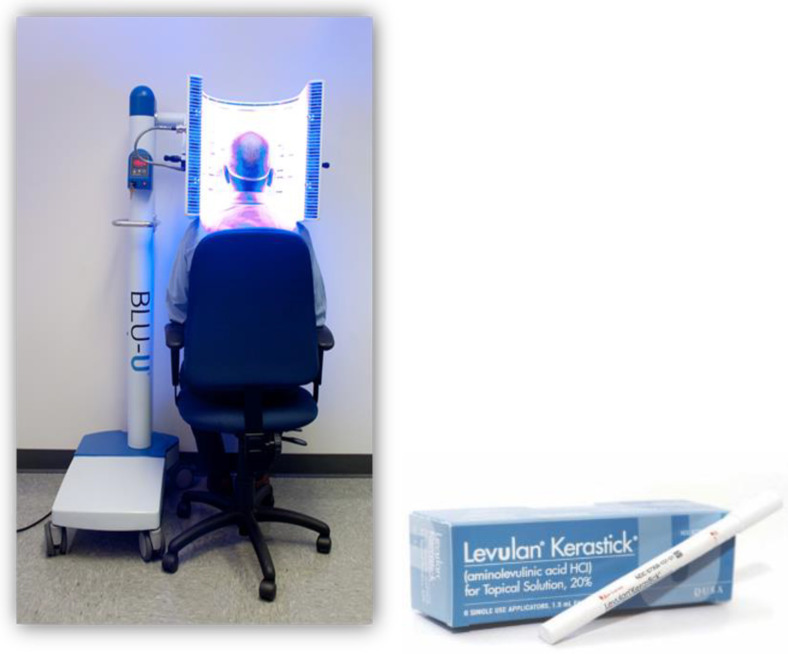
The blue light +20% ALA hydroalcoholic solution applicator ALA PDT system approved by the FDA for the treatment of actinic keratoses of the face and scalp in 1999 [12].

Blue light is 20- to 40-fold as efficient at activating PpIX as red light, because it activates the strongest PpIX absorption band (Figure 3). The first approved commercial light source for topical ALA PDT consisted of U-shaped blue fluorescent lights generating a uniform 10 mW/cm^2^ over the entire face or scalp and needed only 10 J/cm^2^ total light energy to provide a high degree of clinical efficacy. That device, together with a 20% ALA (*w*/*v*) in hydroalcoholic solution applicator, was FDA-approved in 1999 (Figure 4) and to date has been used in over 4 million treatments.

### 2.1. PpIX Photodynamic Diagnosis (PDD)—The Next Step in the Evolution toward Sonodynamic Therapy

**Figure 5:**
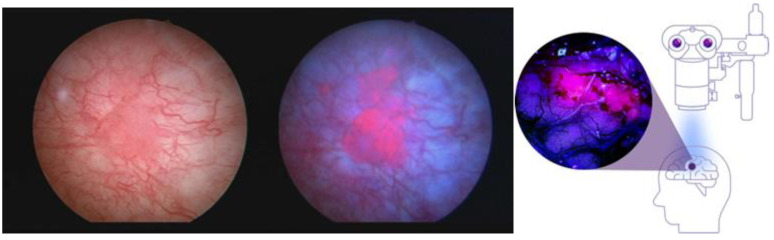
Fluorescent PpIX in the bladder and brain. Intraoperative fluorescence images of bladder carcinoma (left side shows brightfield, middle shows under blue light) and high-grade gliomas (right image). Despite the different forms of ALA used, the colors of the tumor images are interchangeable because they are both from PpIX fluorescence and the activating light wavelengths are both within the blue-violet spectrum. Left image courtesy of Photocure©.

Application of or exposure to excess exogenous ALA for certain cancers of epithelial origin causes such selective accumulation of PpIX, compared with the surrounding normal tissue, that the PpIX fluorescence can be used as a cancer-specific marker for its detection [13,14,15]. Jichlinski [13] instilled 3% ALA intravesically into the urinary bladders of patients with bladder carcinoma, and in 6–7 h could show selective fluorescence in those cancers. In 2010, a hexyl-aminolevulinic acid ester (Cysview^®^) was FDA-approved together with blue light cystoscopy as an optical imaging agent for intravesical use in the cystoscopic detection of carcinoma of the bladder [14]. Stummer and colleagues [15] found that oral dosing of ALA at a concentration of 20 mg/kg caused selective PpIX fluorescence to such a degree by high-grade malignant gliomas, such as glioblastoma, that the fluorescence could be used to guide the neurosurgical tumor resection process. The fluorescence was detected by surgical fluorescence detection microscopes with special filters. The technique was approved by the EMA as an aid to increase resected tumor volume in 2007 [16]. FDA approval took another 10 years and additional clinical trials, and it was approved in 2017 “as an adjunct for the visualization of malignant tissue during surgery” [17]. Figure 5 shows the fluorescence images of bladder carcinoma and high-grade gliomas, illustrating that identical PpIX fluorescence occurs in these tumors following ALA administration.

### 2.2. Interstitial ALA PDT (iPDT) for the Treatment of Recurrent Glioblastomas (rGBMs)

**Figure 6:**
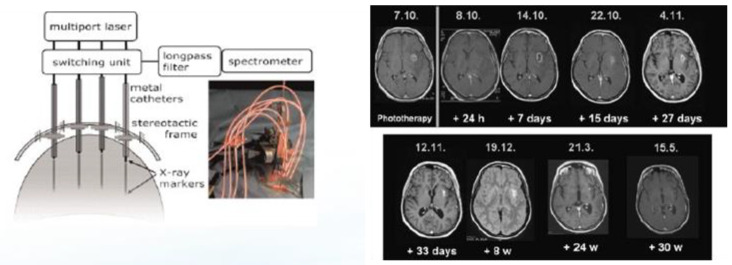
Apparatus and delivery of iPDT to a patient with rGBM. The left image depicts four fiber optics with cylinder-tipped light diffusing tips carry red laser light from a multiport diode laser through a craniotomy into the body of the tumor [19]. The right image shows the course of a single contrast enhancing rGBM over time following a single iPDT treatment [18].

Stummer and colleagues, after developing PDD to aid in the resection of high-grade gliomas, initiated studies in 2007 to activate the PpIX within the tumors with light by inserting up to four cylinder diffuser-tipped fiber optics into the tumor via craniotomy [18,19,20]. The process is invasive and complex, and when performed optimally, it also seeks to quantitate PpIX fluorescence within the target tumor tissue both prior to and after activation with red 635 nm light from a diode laser (Figure 6). Optimal photodynamic activation is thought to result in the photobleaching of PpIX [19] and has been reported in rGBM tumors as large as 10 cc. The apparatus and radiologic results of a single iPDT treatment of an rGBM over time are shown in Figure 6. Despite the invasiveness of the procedure and the delivery of laser light for PpIX activation, no evidence of PDT-induced complications or off-target or side effects such as cerebral edema or hemorrhage have been described in papers from the single site that pioneered the procedure.

In a more recent clinical report on salvage iPDT performed on a total of 44 patients, complications after iPDT were seen in 18 patients (40.0%), who experienced transient worsening of their preexisting neurological deficits. One patient developed malignant edema and underwent emergency decompression within 24 h after iPDT treatment. After six weeks, most deficits resolved or would not inhibit activities of daily life (*n* = 9, CTCAE1). Three patients (6.8%) suffered from residual deficits; in one case, self-care was affected (CTCAE3) [20]. 

Reported outcomes were that the median overall survival (OS) from the first tumor diagnosis was 39.7 months (range 9.8–199.0 months). The median time between first diagnosis and salvage iPDT was 16.9 months (range 3.5–192.4 months). Two years after salvage iPDT treatment, 11 (25%) of the patients were alive, seven (15.9%) of them recurrence-free. In this study, the authors reported that no influence of molecular markers such as MGMT and IDH on the response to iPDT, with or without adjuvant therapy including temozolomide, could be observed. The authors also noted that “...cell death mechanisms...in a patient treated with iPDT are expected to be very heterogeneous, as both light distribution and photosensitizer distribution are not homogeneous” [20].

The long-term survival of a significant percentage of patients after salvage iPDT definitely warrants further investigation. However, despite its introduction as a neurosurgical technique in 2007, iPDT remains a very complex and invasive procedure.

## 3. From PDT to SDT—Preclinical Studies in Rodent Models on SDT for Malignant Gliomas

The goal of SDT using ALA is to take advantage of the specific accumulation of PpIX by gliomas, and for noninvasive ultrasound to provide photons to activate the photodynamic effect through mechanisms such as sonoluminescence. Umemura in 1990 was the first to obtain in vitro evidence of SDT-induced cell death in a murine sarcoma cell line using hematoporphyrin as a sonosensitizer [21]. This research was also groundbreaking in that (a) it provided evidence that a reactive oxygen quencher (histidine) inhibited cell death, supporting evidence for the photodynamic effect, and (b) a sonoluminescence spectrum (300 to >500 nm wavelengths) was produced under the acoustic conditions that resulted in cell death. The output spectrum of sonoluminescence activates almost the entire absorption spectrum of PpIX (Figure 3). It would take more than 10 years for SDT using ALA as sonosensitizer to be applied to murine glioma models.

Jeong et al. reported in 2012 that high-intensity focused ultrasound energy applied via craniotomy directly to the brain surface in the presence of ALA could induce an ALA PDT-like effect in a C6 rat glioma model [22]. The SDT (ALA + MRgFUS)-treated animals showed significant slowing of tumor growth, with the 6 h ALA incubation period (aligned with the highest PpIX concentration) showing greater tumor growth inhibition than the 3 h incubation period. No controls survived past 14 days, while the SDT-treated animals survived to sacrifice at 31 days (24 days post-SDT). Normal brain tissue was not affected.

The first study to show convincingly that the sonodynamic effect could be induced noninvasively (through the intact skull) was that of Suehiro et al., which applied ALA SDT in a mouse glioma model system [23]. Their analysis showed that SDT induced apoptosis and reduced proliferation in the focus and perifocus areas, and necrotic cells in the focus areas only. Administration of edaravone, a reactive oxygen inhibitor to cells in culture, completely blocked the cytotoxic effects of ALA SDT and ultrasound alone. ALA SDT therefore appears to mimic the photodynamic effect, hence the use of the term sonodynamic. Three courses of treatment with ALA SDT significantly reduced the rate of growth of the tumors and prolonged the survival of glioma-bearing mice compared with controls [23]. Using the F98 rat glioma model and the Exablate 4000 Type-2 220 kHz device, Yoshida et al. [24] observed similar effects of reduced proliferation, invasion, and angiogenesis, increased apoptosis, and the absence of tissue damage. Interestingly, the group given ALA alone, without MRgFUS, showed an increased rate of tumor growth compared to the control group, which was not exposed to ALA or MRgFUS. This has not been seen in any other malignant glioma model systems and may be an artifact of this tumor model. The MRgFUS group alone, without ALA, also showed a significant reduction in the rate of tumor progression compared with the ALA group. Although in this study the ALA SDT group shows a significantly slower rate of tumor growth than the MRgFUS group at day 16, there were no significant differences between the growth rate of the two groups by day 23. Furthermore, there were reportedly no differences in animal survival rates among the groups in this study, which differs from both the mouse glioma model and the C6 rat glioma model, albeit with different sonication regimens.

### 3.1. Optimization of Single-Treatment ALA SDT in a C6 Rat Glioma Has Positive Effects on Survival

**Figure 7:**
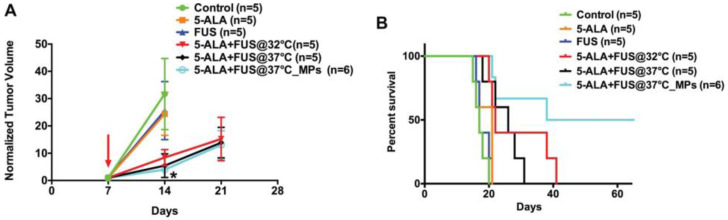
ALA SDT improved survival in a C6 rat glioma model [25]. (**A**) Normalized Treatment performed 7 days after tumor implantation. Control (no treatment), ALA, FUS, ALA + FUS @ 32 °C, ALA + FUS@37 °C and ALA + FUS@37 °C with multi-point sonication (MPS), where instead of sonication applied to a single point in the tumor for 20 min, it was distributed over a grid of 16 points, with each point receiving 75 s sonication. Regardless of baseline temperature, ALA SDT markedly inhibited tumor growth as compared to the control, ALA alone, and FUS alone. Data are presented as mean ± SD. * Represents *p* < 0.05. (**B**) Kaplan–Meier survival curves of tumor-bearing rats. SDT significantly increased the lifespan of tumor-bearing rats, *p* < 0.01.

Hynynen and colleagues sought to optimize the parameters of a single ALA SDT treatment in a C6 rat glioma model [25]. The tumor growth response for animals receiving ALA alone, MRgFUS alone, ALA + MRgFUS, or in the sham control group were evaluated with MRI every week following treatment. While the other groups received sonication to a single tumor focus, the multi-point sonication group received sonication to a grid of 16 areas of the tumor over the 20 minutes of sonication. Tumor growth inhibition and survival were significantly improved in the ALA + MRgFUS group, with 32 °C or 37 °C as the starting core body temperature, compared to ALA or MRgFUS alone (Figure 7). The greatest degree of survival was observed with the multi-point sonication group, indicating that in transferring the procedure to human rGBM patients, multipoint sonication can be safely used within the tumor without endangering normal brain tissue.

Histologic analysis of the tissue effects in this study showed results similar to that of Suehiro et al. [19], with a combination of necrosis and apoptosis and a reduction in the mitotic proliferation index at the area of beam focus. It should be noted that, using the multi-point sonication method, this single-treatment study produced the longest survival times of any animal model glioma study at that time [25].

### 3.2. DIPG (DMG) Tissue Culture Cells Accumulate PpIX When Exposed to Exogenous ALA

The pons is seldom if ever subjected to neurosurgical procedures other than biopsy, so there is no published surgical data showing DIPG fluorescence after Gleolan^®^ dosing. For that reason, we collaborated with Dr. Javad Nazarian’s laboratory at the University of Zurich Children’s Hospital to see if, as predicted, the fast-growing DIPG cells would accumulate PpIX compared with control C6 rat glioma cells [26].

**Figure 8:**
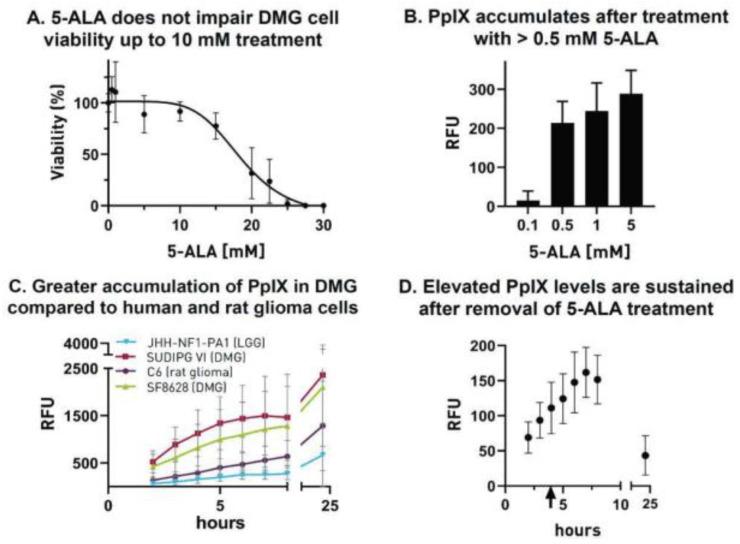
Exposure to exogenous ALA induces robust and sustained PpIX accumulation in DMG primary cultures without reducing viability [26]. (**A**) Viability of DMG cells is maintained up to 10 mM ALA. (**B**) Incubation of 0.5 mM ALA is sufficient to lead to PpIX accumulation. (**C**) Incubation of ALA induces higher PpIX accumulation in DMG cells compared to rat and human glioma cells. (**D**) Elevated PpIX levels persist for at least 8 h in culture following removal of ALA.

The results of this preclinical study (Figure 8) showed that DMG (DIPG) cell growth was not inhibited by concentrations of ALA up to 10 mM (Figure 8A). No fluorescence was observed at 0.1mM 5-ALA concentration, but significant fluorescence was observed at a concentration of 0.5mM and increased to the maximum tested value of 5mM (Figure 8B).The rapidly growing DMG cells also accumulated PpIX faster than the positive control C6 rat glioma tissue culture cells and human low-grade glioma cells (Figure 8C). Furthermore, the elevated PpIX levels in the cultured DIPG cells persisted for more than 8 h after removal of ALA from the medium (Figure 8D) [26]. These results supported the initiation of a clinical trial to treat DIPG tumors in children, and the Children’s National Hospital, with Dr. Roger Packer as the Principal Investigator, was chosen as the first site in this first-in-child treatment study. SDT has not been evaluated in animal models of DIPG.

## 4. Current SDT Clinical Trials

### 4.1. First-in-Man Phase 0/1 Clinical Trial of SDT

An intravenous (iv) formulation of ALA (SONALA-001, SonALAsense Inc., Oakland CA, USA) was developed for use in SDT clinical trials, as i.v. administration avoids first-pass liver metabolism and the gastrointestinal toxicities observed with oral ALA (i.e., Gleolan). SONALA 001 was designed to be given via i.v. bolus, which leads to a rapid increase in plasma concentration of ALA, uptake into cells, and conversion and accumulation of PpIX in glioma cells, thus sensitizing them to SDT treatment.

The first-in-adult human SDT study using SONALA-001 was performed at the Ivy Brain Tumor Center in Phoenix, AZ, and was a Phase 0/1, non-interventional study in patients where resectable recurrent high-grade gliomas of up to 20 cc volume were accrued. Six to seven hours after SONALA-001 infusion (10 mg/kg), patients received MRgFUS with the Exablate 4000 Type 2.0 (220 kHz) device to one-half of their tumor volume, with cohorts of increasing energy of 200 J, 400 J, and 800 J. Four days after SDT treatment, the tumor was resected, and the SDT-treated and control halves of each tumor were compared for biomarkers of lipoperoxidation through 4-hydroxynonenal (4HNE) and for apoptosis via detection of cleaved caspase-3 (ClCas3) [27].

The results are yet to be published; however, the data were presented at the 2023 Annual Society for Neuro Oncology (SNO). In 10 patients, ClCas3 and the oxidative stress biomarker for lipid peroxidation, 4HNE, were significantly increased in SDT-treated tumor tissue vs. control tissue. Levels of reduced thiols (glutathione, cysteine, total thiols) were significantly decreased only in SDT-treated tumor tissue, indicating the production of reactive oxygen species during the SDT treatment. These data reproduce, in human rGBM, the biomarker data observed in animal glioma models successfully treated with ALA SDT [22,23,24,25]. This study is continuing to recruit and to treat patients.

### 4.2. Ongoing Phase 1/2 Clinical Trials of ALA SDT for Malignant Glioma

Beyond disease setting and trial design, the current clinical investigations of SDT for malignant gliomas vary in ALA formulation and delivery route, as well as in the type of ultrasound-generating devices and sonication parameters employed to activate tumor-localized PpIX. Table 1 summarizes the key details of the current neuro-oncology trials for SDT. Four of the six studies listed in Table 1 utilize the Exablate 4000 Type 2.0 (220 kHz) device (Insightec Inc., Haifa, Israel) to deliver MRgFUS to specific target regions based on neurosurgical planning. A Phase 1/2 multicenter, open label, dose escalation and expansion study of SDT with SONALA-001 in combination with the Exablate 4000 Type 2.0 device is being evaluated in subjects with progressive or recurrent GBM and is sponsored by SonALAsense Inc. (NCT05370508). This trial’s main objectives are to evaluate the safety, dose-limiting toxicities, and recommended Phase 2 dose (RP2D) for the study’s expansion portion. This study has a Bayesian 3 + 3 design for escalating SONALA-001 (5 and 10 mg/kg) as well as increasing MRgFUS energy levels (12, 24, and 28 J/subspot). This protocol was amended to allow monthly treatments, with a maximum of 12 treatments. 

The Exablate 4000 Type 2.0 is also being used to activate PpIX in a study entitled “Sonodynamic Therapy with Exablate System in Glioblastoma Patients (Sonic ALA)” being carried out in Italy at the Fondazione I.R.C.C.S. Istituto Neurologico Carlo Besta by Dr. Francisco Prada (NCT04845919). The goal of this Phase 1, single-center, open-label, single-arm study, is to evaluate the safety and feasibility of SDT with oral ALA in patients with newly diagnosed cerebral GBM. The primary objective is safety, with attention to cerebral hemorrhage, edema, or neurological deterioration. Patients able to undergo resection between 15–21 days post SDT have their tumor tissue analyzed for biomarkers of cell death.

There are two additional ongoing clinical trials of SDT in recurrent gliomas that use oral ALA and devices other than the Insightec device to deliver ultrasound energy. One, at the University of Virginia, is an investigator-initiated trial using oral ALA (Gleo-lan) with a neuronavigation-guided FUS device from Navifus Inc (Taipei City, Taipei). (NCT06039709). In this study, patients receive a single SDT treatment targeting a maximum of 50% of the tumor volume (6–20 cc) administered 1–3 weeks before surgery for recurrent GBM. The primary endpoint is the safety and feasibility of this approach as well as analyzing resected tissue for evidence of cell death. The second clinical trial is unique in that it uses a device, CV01, to spread ultrasound diffusely over a large area of the brain after ALA dosing; it is sponsored by Alpheus Medical, Inc. (Oakdale MN, USA) (NCT05362409). Part A of this study will escalate the duration of CV01 ultrasound, followed by the Part B expansion portion, which will further explore safety and tolerability at the recommended duration of CV01 ultrasound. The study is open to patients with supratentorial tumors only, both grade 3 and 4 astrocytomas as well as grade 3 oligodendrogliomas in Part A. Patients with infratentorial or brain stem tumors are excluded from this study.

### 4.3. First-in-Child Pediatric SDT Trial for Diffuse Intrinsic Pontine Glioma (DIPG)

DIPGs are rare and deadly pediatric brain tumors, a subset of diffuse midline gliomas (DMGs) anatomically restricted to the pons. They are extremely aggressive tumors that arise in the pons of the brainstem, which controls many of the body’s most vital functions, such as breathing, blood pressure, and heart rate. Approximately 300 children are diagnosed with DIPG each year in the US, usually between the ages of 5 and 9. There is no currently effective means of treatment for DIPG, and the median survival is less than 1 year after diagnosis. The location of DIPGs in the pons makes it impossible to surgically remove them. Dozens of clinical trials with chemotherapy have failed to improve overall survival in children with DIPG. The current standard of care consists of conventionally fractionated radiotherapy (50.4–59.4 Gy in 28–33 fractions of 1.8 Gy daily, over 6 weeks) and supportive care [28]. Radiotherapy confers a short period of clinical improvement and survival benefit, but it has multiple side effects (nausea, vomiting, headache, fatigue, hearing loss, hair loss, and memory and speech issues) that compromise the quality of life during the short remaining lifespan of children affected by DIPG. Taken together, there is a significant clinical unmet need to develop an effective therapy for DIPG. To address this need, SonALAsense is using SDT as a non-invasive treatment for DIPG. 

**Figure 9:**
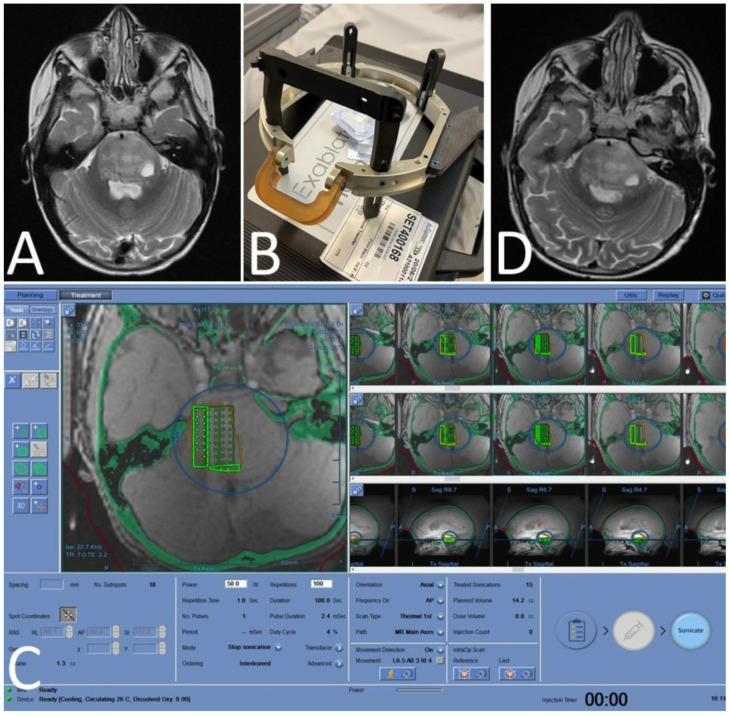
Sonodynamic therapy to the right half of the pons for DIPG in a 5-year old-child [29]. (**A**) pre-procedure MRI demonstrating diffusely infiltrating lesion in the pons; (**B**) focused ultrasound headframe; (**C**) planning station demonstrating sonication targets; (**D**) post-procedure MRI demonstrating no adverse changes.

SonALAsense Inc. is sponsoring a Phase 1/2, multicenter, open-label, dose-escalation and expansion study of SDT with SONALA-001 in combination with the Exablate 4000 Type 2.0 MRgFUS in children with DIPG (NCT05123534). The primary objectives of this trial are to evaluate the safety and tolerability of SDT and to determine the maximum tolerated dose (MTD) or recommended Phase 2 dose (RP2D) of MRgFUS energy in combination with SONALA-001. Patients are accrued in this study following standard of care radiation therapy. A description of that first patient treatment, which treated the entire right half of the pons in a 5-year-old child after she received a 5 mg/kg body weight i.v. dose of SONALA-001, was published as a Technical Communication [29]. Treatment was staged at that time for half of the pons on Day 1, followed by treatment of the second half of the pons four weeks later, to make certain that complications could be avoided. The child received treatment to the second half of her pons four weeks later at the 200 J energy level (Figure 9). No acute or delayed complications or adverse events were observed related specifically to the SDT treatment. To date, five DIPG patients have received SDT treatment to their entire pons in this clinical trial, and treatments are no longer staged to give treatment to each half of the pons separately. All patients receive SDT treatment to their entire pons, and the frequency of treatments has been increased to monthly intervals. SonALAsense is continuing to accrue patients in this study and is expanding to multiple clinical trial sites.

## 5. Likely Regulatory Path for US Commercial Clinical Development of ALA iPDT and ALA SDT in Neuro-Oncology: Potential Barriers

In the US, the FDA views the drug + device combinations used in PDT as a combination product, and the final product of PDT commercial clinical development is a drug label (package insert), which contains instructions on the use of the light source and light delivery device(s) [10,13,30]. The Drug Division (CDER) is the primary regulatory reviewing body of the New Drug Application (NDA) for PDT products, with the Device Division (CDRH) providing device review input of the Pre-Marketing Application (PMA) for the device, which may be included as part of the NDA submission. It is likely that this regulatory process will also be followed for the commercial clinical development of SDT in the US.

For the ALA iPDT of rGBM, a commercial clinical developer might follow the development process that led to the FDA approval of iPDT use of the intravenous preformed Photofrin^®^ (porfimer sodium) PDT photosensitizer together with its laser light source and fiber optic light delivery devices. iPDT with porfimer sodium is indicated for the palliation of patients with completely obstructing esophageal cancer and for reduction of obstruction and palliation of symptoms in patients with completely or partially obstructing endobronchial non-small-cell lung cancer. The drug label contains complete instructions for intravenous administration of the drug as well as the power and energy of laser light to be used and the method of delivery [30]. Dr. Stuart Marcus led the team that created and executed the clinical protocols leading to FDA approval; the regulatory process is fully documented in [31]. Because the patients with completely obstructing esophageal cancer had no recourse to any other treatment, only 17 patients were required for FDA approval. A follow-up confirmatory randomized study enrolling 211 patients was required to obtain FDA approval.

The ALA iPDT for rGBM is a considerably more complex process than iPDT with porfimer sodium, in which a drug is administered and a single fiber optic is placed in the tumor and used for laser light delivery. For optimal ALA iPDT of rGBM, four fiber optics are routinely placed within each tumor, and quantitative fluorescence determination of the target tissue PpIX content should be performed [19,20]. A recent clinical trial report on 44 patients treated small, unifocal, and circumscribed malignant glioma recurrences with a maximum extension of 3 mm, and the authors described the procedure as “technically demanding”. Interpretation of the results of that study are complicated by the fact that nearly half of the patients went directly from iPDT to chemotherapy and radiotherapy. In the United States, the FDA does not consider overall response rates in rGBM to provide sufficient evidence of efficacy and prefers demonstrable improvement in overall survival (OS) for marketing approval of therapies for malignant gliomas.

Johannsson et al. conducted a small single-center study of iPDT in five patients, where patients achieved an objective tumor response rate (ORR) of 3/5 (60%), and all responding patients survived more than 2 years post iPDT [19]. If a clinical commercial developer could optimize ALA iPDT and could duplicate this response in a much larger population (at least 50 patients), the FDA would undoubtedly respond very positively and would work with that developer to accelerate regulatory approval. Other complicating factors would involve the need to perform dose ranging of both light energy and drug dose in a Phase II study using at least three drug doses and three light energy doses at a fixed laser power; it should be noted that there is a standard method of carrying out this type of a study in PDT clinical development as well as standard measures of light power and energy for this type of therapy.

### 5.1. SDT: Suggestions for Commercial Clinical Development in the Treatment of Gliomas

Preclinical and early clinical results in SDT are approximately at the same stage as PDT was in the late 1980s. From lessons learned during the development of porfimer sodium and ALA PDT to FDA approval, we provide the following suggestions for the growing community of SDT clinical researchers in the field of neuro-oncology.

Standardize the presentation of SDT sonication parameters:

- In the US, the drug label will likely carry sonication treatment methods.
- Clearly define device frequency, power, the nature of and frequency of pulses, and some acoustic parameter(s) as well as total energy delivered.
- Determine, with the FDA, the type of sonication parameters required for inclusion in the label and standardize those parameters across SDT clinical trials, regardless of the type of device used to produce ultrasound energies.

When carrying out initial clinical trials, as with PDT, the study design should be a grid of at least three sonosensitizer concentrations and at least three increasing energies of sonication.

### 5.2. Future Research—Elucidating the Short-Term and Long-Term Cellular Effects of ALA iPDT and ALA SDT on Malignant Gliomas: How Many Ways Can You Kill a Cell?

Photonic activation of PpIX and production of reactive oxygen results in short-term cell death, which originally was thought to be limited to apoptosis and necrosis in preclinical models [18,19,20]. The recent work by Sanai et al. [27] corroborated the preclinical work in humans in the first Phase 0 study, showing that within 4 days of a single SDT treatment, increased lipoperoxide liberation and apoptosis in rGBM tissue were observed. However, no radiologic change was observed during that time in human rGBM. Indeed, a single iPDT rGBM treatment most often shows no acute radiologic changes or post-PDT inflammation, but in responding tumors, after a period of 1–2 months, the contrast-enhancing portion of the tumor begins to grow smaller and may take up to 6 months to completely fade away [18,20]. The question remains: which additional mechanism(s) of cell death might account for such a gradual disappearance of a formerly aggressive cancer cell?

This review is too focused to allow a full discussion of all the types of genetically encoded regulated cell death (RCD); however, the types were recently enumerated and well discussed in the recommendations of the Nomenclature Committee on Cell Death [32]. They include intrinsic apoptosis, extrinsic apoptosis, mitochondrial permeability transition (MPT)-driven necrosis, necroptosis, ferroptosis, pyroptosis, parthanatos, entotic cell death, NETotic cell death, lysosome-dependent cell death, autophagy-dependent cell death, immunogenic cell death, cellular senescence, and mitotic catastrophe. The involvement of RCDs in the ability of PDT to cause cancer cell death was recently reviewed [33]. Specifically with respect to ALA PDT, in addition to apoptosis, pyroptosis may be induced during the PDT process. ALA PDT via production of lipoperoxides also may activate ferroptosis. Activation of RIPK3-dependent cell death (necroptosis) in human glioblastoma LN18 cells was also found to be caused by ALA PDT. Further research is necessary to elucidate the full number of RCDs involved in the long-term response of malignant glioma to ALA iPDT and ALA SDT.

## 6. Conclusions and Future Directions in the Evolution of and Research into Clinical SDT

At this point in time in the development of ALA SDT as a new therapeutic procedure in neuro-oncology, it is important to note that, to date, the safety of SDT appears to be equal to or greater than that of ALA iPDT for the treatment of rGBM [18,19,20]. No procedure-related serious adverse events have been reported [29], and except for the need to monitor patients overnight for regulatory-grade clinical trial safety monitoring, ALA SDT can be developed as an outpatient procedure carried out within a single day. Increases in levels of biomarkers such as apoptosis and lipoperoxide liberation from the SDT-treated tissue support the hypothesis that a PDT-like mechanism resulting in membrane disruption is a likely contributing mechanism of action [27]. However, other mechanisms of cell death are likely to be involved in addition to apoptosis, and additional work on the mechanisms involved will be vigorously investigated.

An additional question to consider for future research is the explanation of the absence of inflammatory processes observed post-ALA iPDT or SDT for malignant gliomas in humans and in animal malignant glioma models. The iPDT process involves actual device insertion and cell death occurring within a few days, yet MRIs taken during the acute post-iPDT or SDT period have shown no signs of inflammation or pseudo-progression [18,19,20,27,29]. Recently, Liu et al. [34] used paired single-cell imaging and RNA sequencing of human GBM surgical specimens from patients who had received 5-ALA prior to surgery to demonstrate that cellular labeling, via PpIX fluorescence, was not specific to neoplastic glioma cells. These results may also be used to provide a hypothesis to explain for the first time why no radiologic signs of an inflammatory response have been observed following either ALA iPDT or ALA SDT for rGBM. It is already well known that ALA is taken up by nonneoplastic cells, such as myeloid cells [35], and that ALA-mediated PDT can kill activating but not resting immunocompetent T cell types, with no effect on dendritic cells [36]. Liu et al. also eloquently demonstrated that, in vitro, microglia can take up PpIX secreted from surrounding neoplastic cells, and our interpretation of the data is that ALA is taken up and converted to PpIX not just by tumor cells but also by immunoreactive cells, which are activated within the tumor microenvironment [37]. Future work specifically analyzing the fate of immunoreactive cells within malignant gliomas following iPDT or SDT could be used to gain support for this hypothesis.

### 6.1. Future Directions in the Evolution of Clinical SDT—How Clinical Indications and Use Will Be Determined by the Type of Device Used to Produce PpIX-Activating Ultrasound

#### 6.1.1. Diffusely Targeting Large Brain Areas for SDT (Alpheus Medical)

The device being developed to deliver SDT treatment in clinical trial NCT05362409 reportedly “targets cancer cells throughout the entire brain hemisphere using low-intensity, large-field ultrasound”. The advantages are that MR imaging is not required for treatment, and it is designed ideally for an outpatient clinic. However, the device is limited to the treatment of supratentorial tumors within a single hemisphere. Bi-hemispheric and infratentorial tumors are excluded from the study, so treatment of most childhood brain tumors could not be done using this device. If found effective, the best potential use for this device might be post-surgically as adjuvant therapy, when the tumor burden would be the lowest.

#### 6.1.2. SDT Using a Neuronavigation-Guided Device (Navifus Device)

There is an advantage in using a neuronavigation-guided device (NaviFUS) for SDT in that MRI time is not required for the actual treatment. The device is contained in a clinic room, and treatment is performed as an outpatient procedure. However, with this device in this protocol, the brain tumor to be treated must be within the treatment envelope of the NaviFUS system, described as 30 mm to 80 mm from the inner skull table. Unless this area is extended to much larger areas of the brain, the usefulness of this type of treatment will be limited.

#### 6.1.3. MRgFUS—Using ALA SDT as a Neurosurgical Tool

The first publicly reported results of the first clinical trials of ALA SDT for malignant gliomas (rGBM and DIPG) have been from clinical trials using the i.v. form of ALA, SONALA-001, and MRgFUS and using the Exablate 4000 Type 2.0 device (Insightec [27] and [29], respectively. Due to the safety of the initial results (no dose-limiting toxicities, no severe adverse events related to the procedures), the frequency of treatment in SonALA-sense-sponsored clinical trials SDT-201 (DIPG) and SDT-202 (rGBM) has been increased to monthly treatments. Due to the rapid cellular effects seen with ALA SDT, neurosurgeons have the ability to adjust and tailor the therapy on a month-to-month basis, based on areas that are responding, are stable, or are slightly progressing. The method of sonication continues to evolve to enable increased treatment volumes with adjusted sonication parameters, to reduce the time needed to treat large areas of the brain. The current ongoing clinical trials using the Exablate 4000 Type 2 device are treating patients with supratentorial, infratentorial, and bi-hemispheric tumors. Therefore, this method has the potential to treat most adult and pediatric brain tumors.

## Figures and Tables

**Table 1:** Current SDT Clinical Trials in Neuro-Oncology.

NCT Number	Study Title	Condition	Description	Treatment	Endpoints
NCT06039709	Sonodynamic Therapy in Patients with Recurrent GBM	rGBM	Phase 1, single center	Single treatment of oral 5-ALA with Neuronavigation-guided LIFU (NaviFUS), 1–3 weeks before surgery	Safety, biomarker analysis
NCT04845919	Sonodynamic Therapy with ExAblate System in GBM Patients (Sonic ALA)	GBM	Phase 2, single center	Single treatment of oral 5-ALA with MRgFUS (Exablate), followed by surgery 15–21 days post SDT	Safety, biomarker analysis
NCT04559685	Study of Sonodynamic Therapy in Participants with Recurrent HGG	HGG	Phase 0, single center	Single treatment of IV ALA (SONALA-001) with MRgFUS (Exablate), followed by surgery 4–6 days post SDT	Safety, biomarker analysis, immune profiling
NCT05362409	Study to Evaluate 5-ALA Combined with CV01 Delivery of Ultrasound in Recurrent HGG	HGG	Phase 1, multicenter	Monthly treatment with oral ALA with CV01 ultrasound	Safety, determination of Maximum Tolerable Duration of Sonication (MTDu)
NCT05370508	A Study of Sonodynamic Therapy Using SONALA-001 and Exablate 4000 Type 2.0 in Subjects with Recurrent GBM	rGBM	Phase 1/2, multicenter	Monthly treatments of IV ALA (SONALA-001) with MRgFUS (Exablate) device	Safety and tolerability, determination of MTD and RP2D
NCT05123534	A Phase 2 Study of Sonodynamic Therapy Using SONALA-001 and Exablate 4000 Type 2.0 in Patients With DIPG	DIPG	Phase 1/2, multicenter	Monthly treatments of IV ALA (SONALA-001) with MRgFUS (Exablate) device	Safety and tolerability, determination of MTD and RP2D

DIPG—diffuse intrinsic pontine glioma; HGG—high-grade glioma; GBM—glioblastoma; LIFU—low-intensity focused ultrasound; MRgFUS—MR-guided focused ultrasound; MTD—maximum tolerated dose; MTDu—maximum tolerated duration; rGBM—recurrent glioblastoma; RP2D—recommended phase 2 dose.
